# Improving health care response to domestic violence

**DOI:** 10.1192/j.eurpsy.2021.212

**Published:** 2021-08-13

**Authors:** A. Dowrick

**Affiliations:** Nuffield Department Of Primary Care Health Sciences, University of Oxford, Oxford, United Kingdom

## Abstract

**Abstract Body:**

1 in 4 women in Europe will experience domestic violence and abuse (DVA) in their lifetime. Abuse can take many forms, including, psychological, physical, sexual, financial, and emotional abuse. There is growing recognition of the health consequences of DVA in public policy and academic research across Europe and the globe. These include but are not limited to: a negative impact on mental health; increased substance misuse; increased presentation in emergency departments; increased rates of abortion or miscarriage; and increased presence of any sexual health problem. International and national policy guidance indicates that healthcare professionals have important roles in responding to patients experiencing DVA, usually with regard to identifying abuse and referring for specialist support. Barriers to engaging in DVA relate to lack of confidence in recognising abuse or initiating conversations about it, stigma surrounding DVA, fears of exacerbating violence, limited awareness of the resources available to support patients, and limited training in providing trauma-informed medical care. This session will give insight into efforts to improve the healthcare response to DVA. It will draw together examples of healthcare DVA programmes from across the globe and bring together lessons learnt about what works and what does not work in addressing DVA in clinical settings, with specific insights for professionals working in mental health services.

**Disclosure:**

No significant relationships.

